# Supplementary material for: Homochiral antiferromagnetic merons, antimerons and bimerons realized in synthetic antiferromagnets
Source: Nat Commun. 2024 Feb 26;15:1641. doi: 10.1038/s41467-024-45375-z (PMC10897388; doi:10.1038/s41467-024-45375-z)
Supplement: Supplementary file 1 — Supplementary Information [file 41467_2024_45375_MOESM1_ESM.pdf]

Homochiral antiferromagnetic merons, antimerons and bimerons realized in synthetic antiferromagnets

Mona Bhukta[1], Takaaki Dohi[1,2,*], Venkata Krishna Bharadwaj[1], Ricardo Zarzuela[1], Maria-Andromachi Syskaki[1,3], Michael Foerster[4], Miguel Angel Niño[4], Jairo Sinova[1], Robert Frömter[1,*], and Mathias Kläui[1,*]

[1]Institute of Physics, Johannes Gutenberg-University Mainz, 55099 Mainz, Germany

[2]Laboratory for Nanoelectronics and Spintronics, Research Institute of Electrical Communication, Tohoku University, 2-1-1 Katahira, Aoba, Sendai 980-8577, Japan

[3]Singulus Technologies AG, Hanauer Landstrasse 107, 63796 Kahl am Main, Germany

[4]ALBA Synchrotron Light Facility, 08290 Cerdanyola del Vallès, Barcelona, Spain

[*]tdohi@tohoku.ac.jp,froemter@uni-mainz.de, klaeui@uni-mainz.de

ABSTRACT

This is the supplementary information for the article "Homochiral antiferromagnetic merons, antimerons, and bimerons realized in synthetic antiferromagnets"

1 Homochiral antiferromagnetic merons/antimerons in SyAFM platforms

Three-dimensional illustrations of AFM-coupled meron-meron composites with a topological charge of $Q = \frac{1}{2}$ and helicities $\gamma = 0, \frac{\pi}{2}, \pi$, and $\frac{3\pi}{2}$ are shown in panels (a) to (d) in Fig. S1. The black and white colors represent the upward and downward core polarity, respectively, while the IP color map is defined in the top left corner of Fig. S1. Panels (e) to (h) illustrate different AFM antimerons with $Q = \frac{1}{2}$ and helicity values of 0, $\frac{\pi}{2}$, π, and $\frac{3\pi}{2}$, respectively. We note that antimerons show an inverted angular sequence of in-plane color contrast in SEMPA imaging compared to their meron counterparts. Additionally, it should be noted that the solitons in adjacent FM layers exhibit identical winding numbers but opposite core polarities, and their helicities differ by a factor of π. Similarly, AFM merons and antimerons with $Q = -\frac{1}{2}$ and helicities $\gamma = 0, \frac{\pi}{2}, \pi$, and $\frac{3\pi}{2}$ are illustrated in Fig. S2. A bimeron results from the combination of a meron and an antimeron with opposite topological charges.

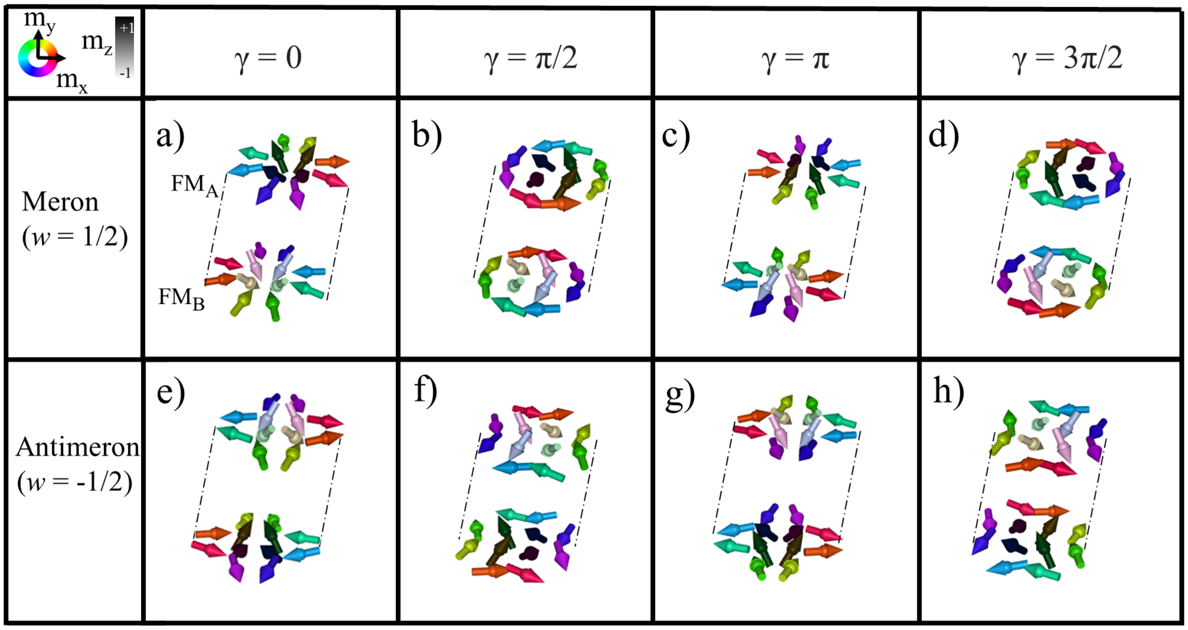

Figure S1. **Spin configuration of AFM merons and antimerons in a SyAFM platform having Q = $\frac{1}{2}$.(a)–(d)** $Q = \frac{1}{2}$ AFM meron with helicities $\gamma = 0, \frac{\pi}{2}, \pi$, and $\frac{3\pi}{2}$, respectively. **(e)–(h)** $Q = \frac{1}{2}$ AFM antimeron with helicities $\gamma = 0, \frac{\pi}{2}, \pi$, and $\frac{3\pi}{2}$, respectively.

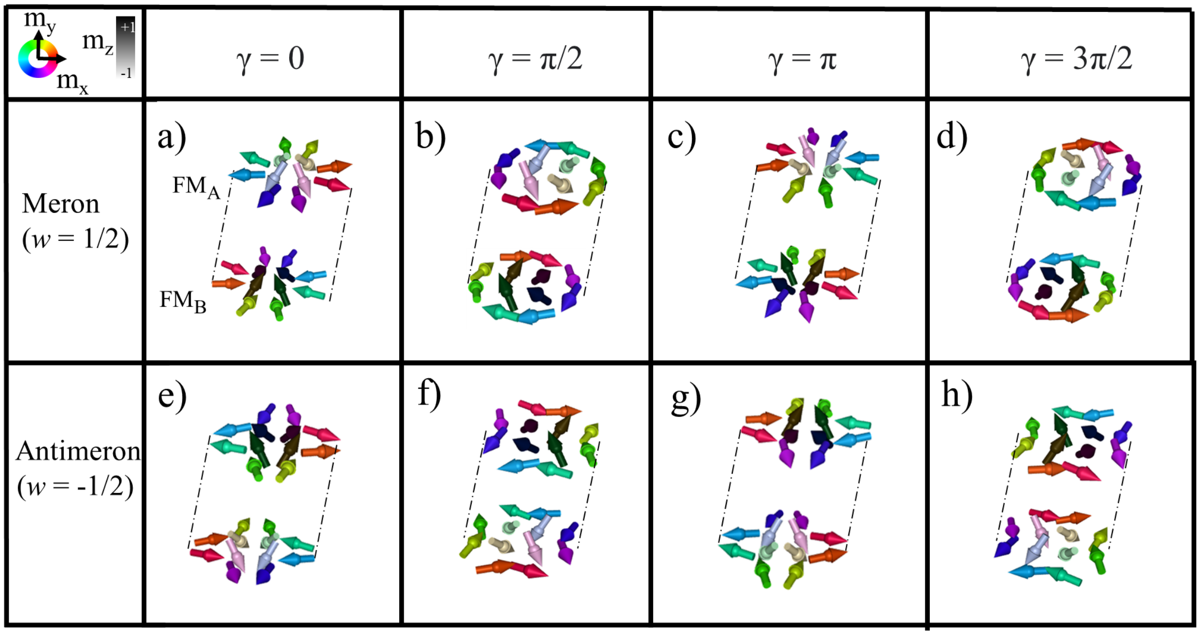

Figure S2. **Spin configuration of AFM merons and antimerons in a SyAFM platform having Q = -$\frac{1}{2}$ (a)–(d)** $Q = -\frac{1}{2}$ AFM meron with helicities $\gamma = 0, \frac{\pi}{2}, \pi$, and $\frac{3\pi}{2}$, respectively. **(e)–(h)** $Q = -\frac{1}{2}$ AFM antimeron with helicities $\gamma = 0, \frac{\pi}{2}, \pi$, and $\frac{3\pi}{2}$, respectively.

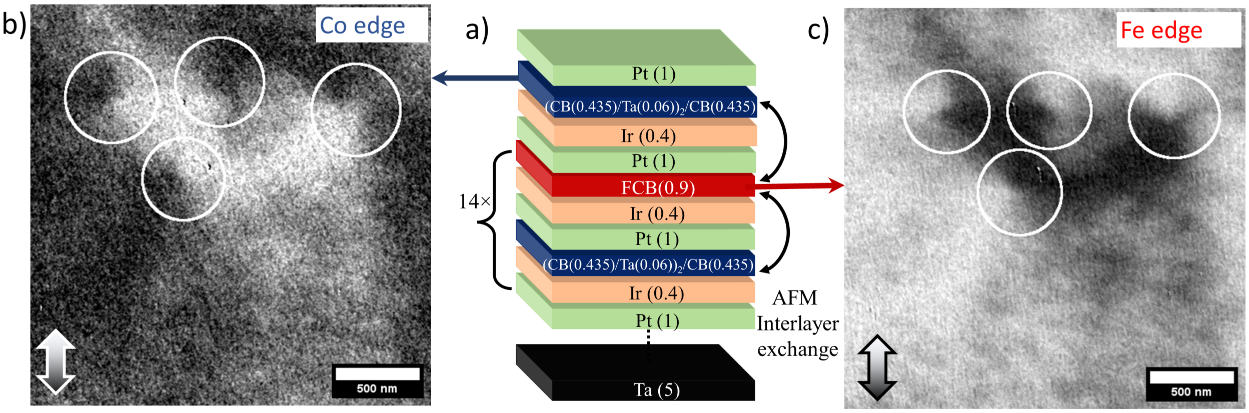

Figure S3. **XMCD-PEEM images of stack** #4 **at Co and Fe edges (a)** Sample structure for stack #4 . XMCD-PEEM images of a meron pattern in the SyAFM **(b)** Co and c) Fe L_3 edge contrast of the same surface area. The IP-sensitive direction is along the horizontal. White circles mark meron spin textures in the top layers of the SyAFM. The observation of an inverted contrast between the Co-rich top and the Fe-rich second FM layer confirm AFM coupling between these layers.

2 Observation of SyAFM merons using XMCD-PEEM

We performed XMCD-PEEM experiments on stack #4 using its element specificity to prove the AFM coupling between the FM layers. XMCD-PEEM images are acquired at the ALBA synchrotron (BL-24 Circe) facility using PEEM, where the beam is incoming at an angle of 16° with respect to the sample surface. Magnetic contrast is obtained by calculating the asymmetry between images taken with right and left-circularly polarized radiation. X-ray absorption (XAS) spectra around the Fe and Co L_3 edges find the maxima at 707.0 eV and 777.6 eV, respectively. Stack #4 has an odd number of layers (29 layers) and is shown in Fig. S3a. We note that this SyAFM is purposefully designed to have an additional FM (CoB) on the top to see a clear contrast at both Co and Fe edges, representing consecutive FM layers. Fig. S3 b,c show XMCD-PEEM images of the same sample area of the SyAFM stack at room temperature, but resonantly tuned to either the Co or Fe L_3 edge. Panels b) and c) show the magnetic contrast at the Co and Fe edges, respectively. By imaging at the Fe edge, we are able to isolate the magnetic contrast from the second magnetic layer, as there is no Fe present in the top one. The centers of the observed meron spin textures are marked with white circles. In contrast, the magnetic contrast at the Co edge originates predominately from the topmost FM layer, as the second layer has effectively less Co. We find that the (anti)merons visible in this top layer are located exactly at the same positions as in the second layer, however, the magnetic contrast is inverted. This confirms the AFM coupling between the domains, domain walls, and the centers of merons and antimerons in the SyAFM system.

3 SEMPA and MFM images for stack #2b

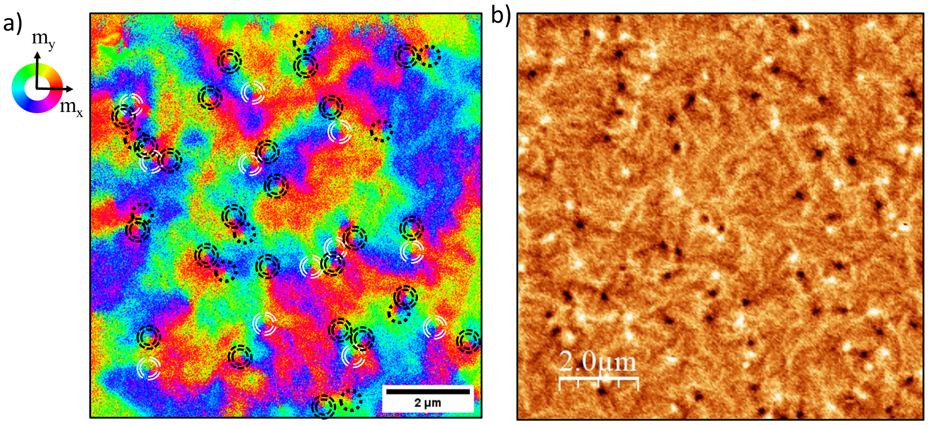

Figure S4. SEMPA and MFM images at different spot of the stack #2b. (**a**) SEMPA image of a meron pattern in the topmost layer of the SyAFM stack #2b. (**b**) MFM image of this pattern from a different area on the same film showing the core polarities. Black single circles and white double circles indicate merons with helicity $\gamma = 0$ and $\gamma = \pi$, respectively. Antimerons are marked using a double black circle.

Fig. S4a shows the domain structure of the SyAFM stack #2b at room temperature and zero magnetic fields. Black single circles and white double circles indicate merons with helicity $\gamma = 0$ and $\gamma = \pi$, respectively. Antimerons are marked using a double black circle. Note that here we find an almost equal number of merons and antimerons from SEMPA images. Fig. S4b demonstrates the OOP contrast of the domain structure of a different part of the same film using MFM.

3.1 Deleting and re-creating (anti)merons

Here we investigate the effect of magnetic fields on the stability of (anti)merons, a class of topological textures present in magnetic materials. Using SEMPA measurements, the sample of stack #2*a* was analyzed at room temperature and under various magnetic field conditions. Fig. S5a depicts the state of the sample after being subjected to out-of-plane demagnetization, revealing the presence of (anti)merons. In contrast, Fig. S5b illustrates the deletion of these meron spin textures upon the application of an in-situ magnetic field of 50mT along the *x*-direction. The application of an in-situ magnetic field of -50mT along the -*x*-direction, as depicted in figure S5c, results in the re-emergence of the (anti)merons in a different magnetic location. Lastly, Fig. S5d shows the spin state after the application of an in-plane oscillating magnetic field. These results demonstrate the ability to manipulate topological spin structures by applying magnetic fields.

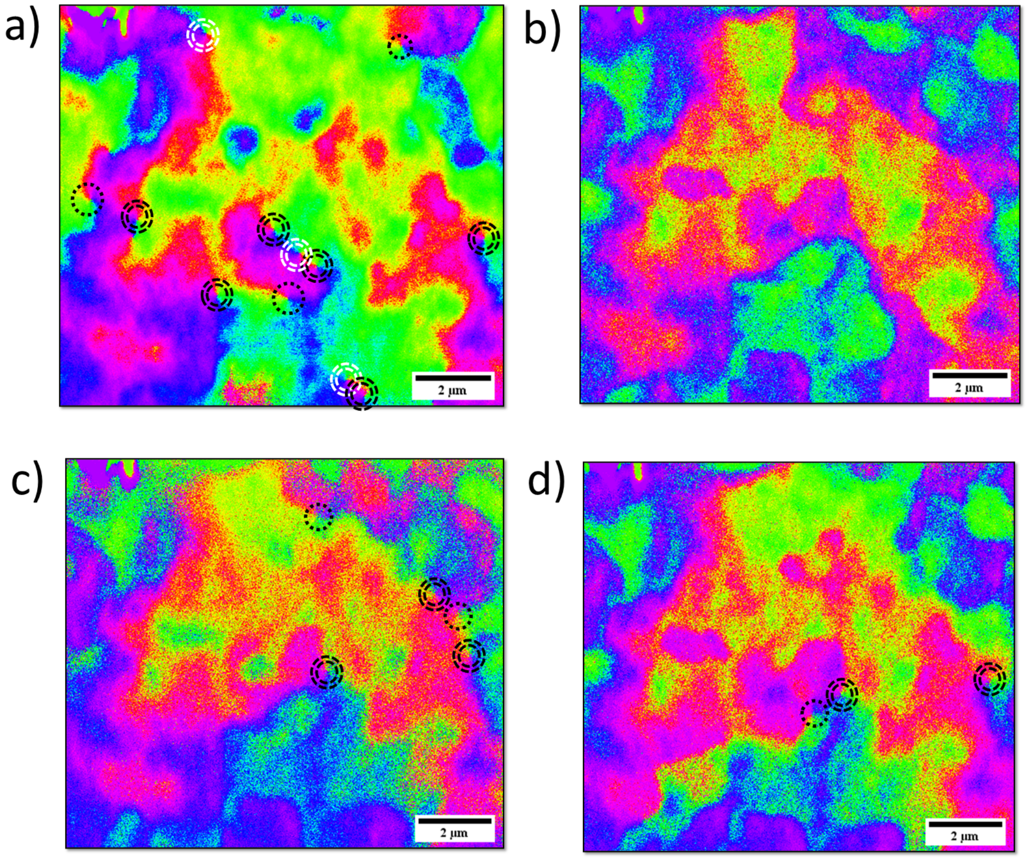

Figure S5. Deleting and re-creating (anti)merons. (**a**) Out-of-plane demagnetized state, (**b**) state after the application of +50mT magnetic field along the x-direction, (**c**) state after the application of -50mT magnetic field along the x-direction, (**d**) in-plane demagnetized state.The demagnetized state is achieved following the application of an oscillating magnetic field. Black single circles and white double circles indicate merons with helicity $\gamma = 0$ and $\gamma = \pi$, respectively. Antimerons are marked using a double black circle.

4 Analytical theory

In this section of the supplementary material we discuss our analytical treatment of a bilayer SyAFM, which we model as a system of two FM layers separated by a metallic spacer, see Fig. S6. The energetics of each FM layer contains all the ingredients needed to stabilize topological solitons, namely (direct) exchange interaction, DM (antisymmetric exchange) interaction, and on-site uniaxial anisotropy. The latter, in turn, contains both PMA and magnetostatic contributions. Furthermore, the itinerant carriers within the spacer mediate an (indirect) interlayer exchange interaction. We note that magnetic dipolar interactions between the two ferromagnetic layers play a relevant role in this type of heterostructures near the SRT point. Consequently, the energy functional describing the bilayer SyAFM in the low-energy long-wavelength limit reads

$$\mathscr{E}[\boldsymbol{M}_A,\boldsymbol{M}_B] = \int_{\mathscr{S}} d^2\vec{r}\left\{\tfrac{A_A}{2}(\nabla\boldsymbol{M}_A)^2 + \tfrac{A_B}{2}(\nabla\boldsymbol{M}_B)^2 + D_A\mathscr{L}_{\rm N}[\boldsymbol{M}_A] + D_B\mathscr{L}_{\rm N}[\boldsymbol{M}_B] - K_A^{\rm eff}M_{A,z}^2 - K_B^{\rm eff}M_{B,z}^2 \right.$$
$$\left. - [\boldsymbol{H}_{\rm d}\cdot(\boldsymbol{M}_A+\boldsymbol{M}_B)] + \delta_{\rm ex}(\boldsymbol{M}_A\cdot\boldsymbol{M}_B)\right\}, \tag{1}$$

where $\boldsymbol{M}_i(\vec{r})$ denotes the macroscopic spin density field of the i-th ferromagnetic layer ($i = \mathrm{A,B}$), $M_{s,i} \equiv |\boldsymbol{M}_i| = \mathrm{ct}$, are the corresponding saturation magnetizations, and the basal plane $\mathscr{S}$ extends in the x and y directions. Here, $A_{A,B}$ and $D_{A,B}$ are the spin stiffness constants and the DM strengths of the FM layers, respectively, and $\delta_{\rm ex}$ represents the interlayer exchange constant, which depends strongly on the interlayer distance. We note that this distance is chosen so that the indirect exchange interaction is antiferromagnetic. The interlayer demagnetizing field $\boldsymbol{H}_{\rm d} = H_{\rm d}\hat{e}_z$ is, by symmetry arguments, directed towards the normal to the heterostructure. Its strength scales as $H_{\rm d} \propto \frac{1}{d_{\rm FM}}$ with the distance $d_{\rm FM}$ between the FM layers. In addition, the effective anisotropy constants $K_{{\rm eff},i} = K_i - \frac{\mu_0}{2}M_{s,i}^2$ ($i = A,B$), split into the magnetocrystalline terms $K_{A,B}$ and the effective magnetostatic contributions $K_{{\rm d},i} \equiv -\frac{\mu_0}{2}M_{s,i}^2$, the latter resulting from the structure $\hat{N} = \mathrm{diag}(0,0,1)$ for the demagnetizing tensor in the thin-film approximation. We note that the fabrication process of the heterostructure is such that each ferromagnetic layer is in the vicinity of the SRT point, namely $K_{\rm eff} \simeq 0$. We note as well that the interplay between the interlayer exchange and magnetic dipolar interactions determines the orientation (IP vs. OOP) of the magnetization fields $\boldsymbol{M}_{A,B}$ in this SRT regime, since the energy scale ascribed to the demagnetizing field $\boldsymbol{H}_{\rm d}$ becomes dominant here. The interfacial DM interaction is described by the Lifshitz invariant

$$\mathscr{L}_{\rm N}[\boldsymbol{M}_i] = \boldsymbol{M}_i\cdot(\tilde{\nabla}\times\boldsymbol{M}_i) = (\boldsymbol{M}_i\cdot\nabla)M_{z,i} - M_{z,i}(\nabla\cdot\boldsymbol{M}_i), \tag{2}$$

with $\tilde{\nabla} = \hat{z}\times\nabla$. This form of DM interaction is well known to induce out-of-plane rotations of the localized spins.

Phenomenologically, we can describe the aforesaid SyAFM as a bipartite magnetic-sublattice system, each sublattice corresponding to one of the ferromagnetic layers. In general (namely, far from the compensation point $\Theta \equiv M_{{\rm s},A}^2 - M_{{\rm s},B}^2 = 0$), this magnetic platform behaves as a ferrimagnet, whose effective long-wavelength theory can be built upon the Néel order $\boldsymbol{L}$ and the total magnetization $\boldsymbol{M}$ fields,

$$\boldsymbol{L} \equiv \boldsymbol{M}_A - \boldsymbol{M}_B, \qquad \boldsymbol{M} \equiv \boldsymbol{M}_A + \boldsymbol{M}_B, \tag{3}$$

which satisfies the holonomic constraints

$$\boldsymbol{M}\cdot\boldsymbol{L} = M_{{\rm s},A}^2 - M_{{\rm s},B}^2 = \Theta, \tag{4}$$
$$\boldsymbol{M}^2 + \boldsymbol{L}^2 = 2(M_{{\rm s},A}^2 + M_{{\rm s},B}^2). \tag{5}$$

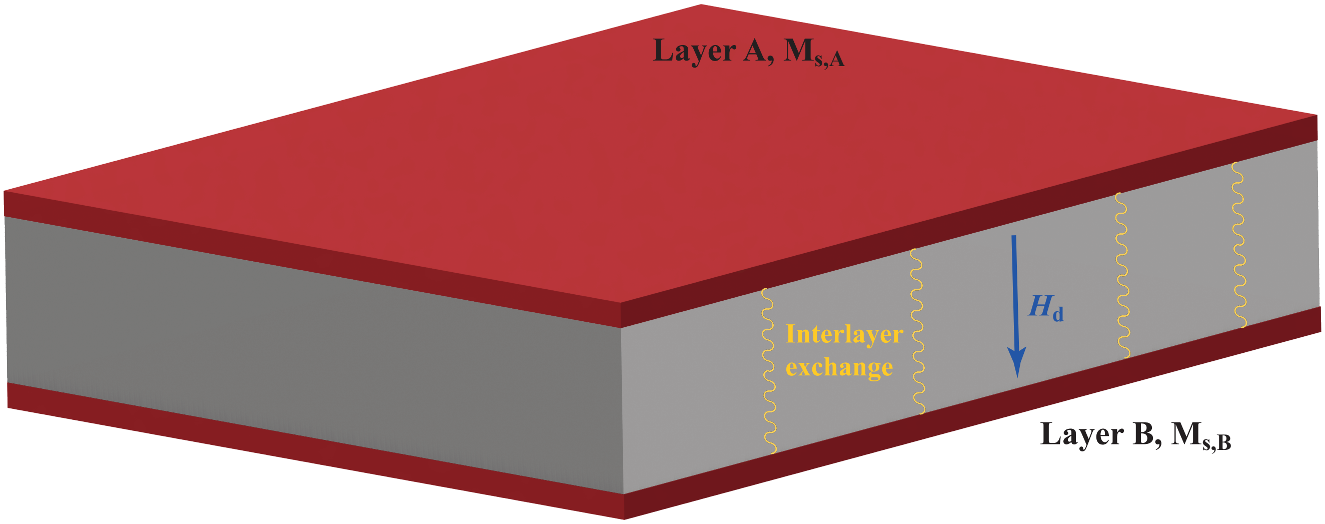

Figure S6. Sketch of the bilayer SAF discussed in the main text. The metallic spacer (grey) is sandwiched in between two ferromagnetic layers (red) which present different saturation magnetizations, $M_{s,A}$ (sub-lattice A) and $M_{s,B}$ (sub-lattice B). The itinerant charges flowing through the spacer mediate the interlayer (antiferromagnetic) exchange interaction. Furthermore, the two ferromagnetic layers also interact via a demagnetizing field $\boldsymbol{H}_{\mathrm{d}}$ along the normal to the heterostructure. An effective low-energy long-wavelength description of the SyAFM can be obtained in terms of its Néel order $\boldsymbol{L} = \boldsymbol{M}_A - \boldsymbol{M}_B$ and macroscopic spin density $\boldsymbol{M} = \boldsymbol{M}_A + \boldsymbol{M}_B$, which corresponds to that of a ferrimagnet (far from the compensation point $\Theta = M_{s,A}^2 - M_{s,B}^2 = 0$).

By inverting Eq. (3) [that is, $\boldsymbol{M}_A = \frac{1}{2}(\boldsymbol{M}+\boldsymbol{L})$ and $\boldsymbol{M}_B = \frac{1}{2}(\boldsymbol{M}-\boldsymbol{L})$] and by incorporating the result into Eq. (1), we obtain the energy functional

$$\begin{aligned}\mathscr{E}[\boldsymbol{M},\boldsymbol{L}] = \int_{\mathscr{S}} d^2\boldsymbol{r} \Big[&\tfrac{A_A+A_B}{8}\left[(\nabla\boldsymbol{M})^2+(\nabla\boldsymbol{L})^2\right] + \tfrac{A_A-A_B}{4}\left[\nabla\boldsymbol{M}\cdot\nabla\boldsymbol{L}\right] + \tfrac{D_A+D_B}{4}\Big[\boldsymbol{M}\cdot(\tilde{\nabla}\times\boldsymbol{M}) \\ &+\boldsymbol{L}\cdot(\tilde{\nabla}\times\boldsymbol{L})\Big] + \tfrac{D_A-D_B}{4}\left[\boldsymbol{M}\cdot(\tilde{\nabla}\times\boldsymbol{L})+\boldsymbol{L}\cdot(\tilde{\nabla}\times\boldsymbol{M})\right] - \tfrac{K_{\mathrm{eff},A}+K_{\mathrm{eff},B}}{4}\left(M_z^2+L_z^2\right) \\ &- \tfrac{K_{\mathrm{eff},A}-K_{\mathrm{eff},B}}{2}M_zL_z - \boldsymbol{M}\cdot\boldsymbol{H}_{\mathrm{d}} + \frac{\delta_{\mathrm{ex}}}{4}\left(\boldsymbol{M}^2-\boldsymbol{L}^2\right)\Big].\end{aligned} \tag{6}$$

Minimization of the above functional with account of the constraints (4) and (5) yields the saddle point equations:

$$\frac{\delta\mathscr{E}}{\delta\boldsymbol{M}} = \frac{\delta_{\mathrm{ex}}}{2}\boldsymbol{M} + \mu_1\boldsymbol{L} + 2\mu_2\boldsymbol{M} - \boldsymbol{H}_{\mathrm{d}} = 0, \tag{7}$$

$$\frac{\delta\mathscr{E}}{\delta\boldsymbol{L}} = -\frac{\delta_{\mathrm{ex}}}{2}\boldsymbol{L} + \mu_1\boldsymbol{M} + 2\mu_2\boldsymbol{L} = 0, \tag{8}$$

where μ_1 and μ_2 are the Lagrange multipliers associated with Eqs. (4) and (5), respectively. We note that in the above equations, we have disregarded other terms contributing at the subleading order to the energetics of the ferrimagnet in the vicinity of both the SRT and compensation points. The expressions for the Lagrange multipliers are therefore

$$\mu_1 = \frac{-\Theta\delta_{\mathrm{ex}}\boldsymbol{L}^2 + (\boldsymbol{H}_{\mathrm{d}}\cdot\boldsymbol{L})\boldsymbol{L}^2}{\boldsymbol{L}^4-\Theta^2}, \qquad 2\mu_2 = \frac{\frac{\delta_{\mathrm{ex}}}{2}\left(\boldsymbol{L}^4+\Theta^2\right) - (\boldsymbol{H}_{\mathrm{d}}\cdot\boldsymbol{L})\Theta}{\boldsymbol{L}^4-\Theta^2}, \tag{9}$$

from which we obtain the following expression for the slave field $\boldsymbol{M}$:

$$\boldsymbol{M} = c_1\boldsymbol{L} + c_3\boldsymbol{L}\times(\boldsymbol{H}_{\text{d}}\times\boldsymbol{L}), \qquad c_1 \equiv \frac{\Theta}{\boldsymbol{L}^2}, \;\; c_3 \equiv \frac{1}{\delta_{\text{ex}}\boldsymbol{L}^2}. \tag{10}$$

The above expression has been derived in the regime $\Theta \ll 1$ (i.e., near the compensation point), so that the total magnetization field is expanded up to first order in the parameter Θ. Next, we incorporate Eq. (10) into the expression (6) and assume that the material parameters for both ferromagnetic layers are the same (i.e., $A_A = A_B \equiv 2A$, $D_A = D_B \equiv 2D$, $K_{\text{eff},A} = K_{\text{eff},B} \equiv 2K_{\text{eff}}$, $\delta_{\text{ex}} \equiv 2\delta$, etc.), which results in the following effective energy functional for the ferrimagnet in terms of the Néel order:

$$\begin{aligned}\mathscr{E}[\boldsymbol{L}] &= \int_{\mathscr{S}} d^2\boldsymbol{r}\left[\tfrac{A}{2}(\nabla\boldsymbol{L})^2 + D\boldsymbol{L}\cdot(\tilde{\nabla}\times\boldsymbol{L}) - K_{\text{eff}}L_z^2 + c_3\left(\boldsymbol{H}_d\cdot\boldsymbol{L}\right)^2 - c_1\left(\boldsymbol{H}_d\cdot\boldsymbol{L}\right) + \tfrac{\delta}{2}\left(c_1^2-1\right)\boldsymbol{L}^2\right]\\ &= \int_{\mathscr{S}} d^2\boldsymbol{r}\left[\tfrac{A}{2}(\nabla\boldsymbol{L})^2 + D\boldsymbol{L}\cdot(\tilde{\nabla}\times\boldsymbol{L}) - \left(K_{\text{eff}} - \tfrac{H_{\text{d}}^2}{2\delta\boldsymbol{L}^2}\right)L_z^2 - \tfrac{\Theta}{\boldsymbol{L}^2}H_{\text{d}}L_z\right].\end{aligned} \tag{11}$$

In the above expression, we have disregarded constant contributions to the energy as well as terms contributing at the subleading order (e.g., terms quadratic in Θ). When the SyAFM reaches the compensation point, namely $\Theta = 0$, we obtain the energy functional

$$\mathscr{E}_{\text{cp}}[\boldsymbol{L}] = \int_{\mathscr{S}} d^2\boldsymbol{r}\left[\tfrac{A}{2}(\nabla\boldsymbol{L})^2 + D\boldsymbol{L}\cdot(\tilde{\nabla}\times\boldsymbol{L}) - \left(K_{\text{eff}} - \tfrac{H_{\text{d}}^2}{2\delta\boldsymbol{L}^2}\right)L_z^2\right]. \tag{12}$$

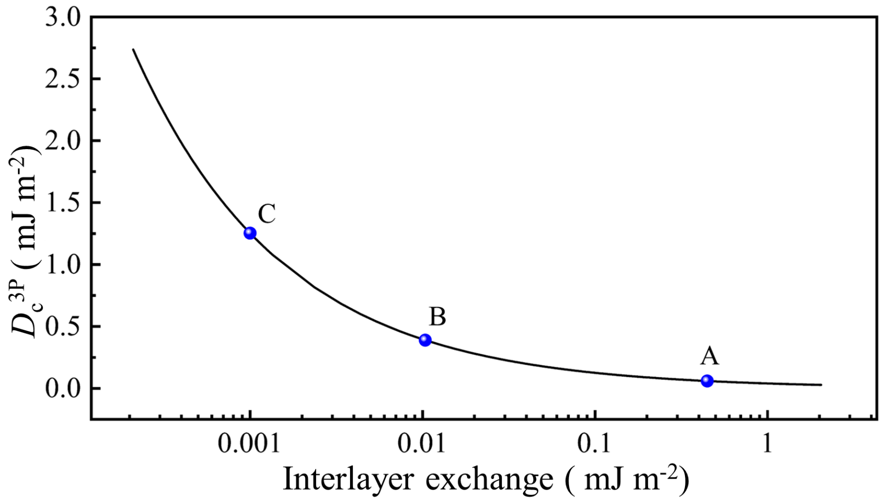

Figure S7. Critical point of bimerons in synthetic antiferromagnets. The blue data points correspond to the phase diagram depicted in Fig. 4, where 2δ takes values of 0.44 mJm^{-2} for case A, 10 μJm^{-2} for case B, and 1 μ Jm^{-2} for case C.

5 Micromagnetic Simulation

We perform micromagnetic simulations to understand the influence of the different magnetic parameters on the antiferromagnetic bimeron properties in a SyAFM using MuMax^3 as mentioned in the method section. The effect of DMI, effective anisotropy, and the strength of interlayer exchange on the separation between the meron and antimeron spin textures constituting the bimeron are discussed in detail.

The influence of the Dzyaloshinskii-Moriya interaction (DMI) on the separation between the two meron spin textures in a bimeron is examined in Fig. S8a. By varying the DMI from (0.14 - 0.34) mJm^{-2} at K_{eff} = 0.1 MJm^{-3} and keeping other parameters constant, our simulation results show that the separation between the meron and antimeron steadily increases with DMI until the system achieves perpendicular magnetization. The increase in DMI leads to the induction of more perpendicular spin components, which raises the demagnetization energy (E_{demag}) and reduces the anisotropy energy (E_{Aniso}). It should be noted that the DMI and perpendicular magnetic anisotropy cooperate to stabilize the bimeron near the spin reorientation transition (SRT) region, unlike a skyrmion. To investigate the separation between the core polarities of the meron and antimeron in a bimeron, we varied the effective anisotropy ($|K_{\text{eff}}|$) in the range of (0.5 - 1.8) $\times$ 10^4 Jm^{-3} for D = 0.25 mJm^{-2}, as presented in Fig. S8b. Increasing $|K_{\text{eff}}|$ results in a decrease in the separation between the meron and antimeron, accompanied by an increase in the total energy (E_{total}) and a decrease in the demagnetization energy (E_{demag}). This behavior can be attributed to the perpendicular magnetic anisotropy, which favors the stabilization of out-of-plane magnetization and results in a larger separation between the meron and antimeron.

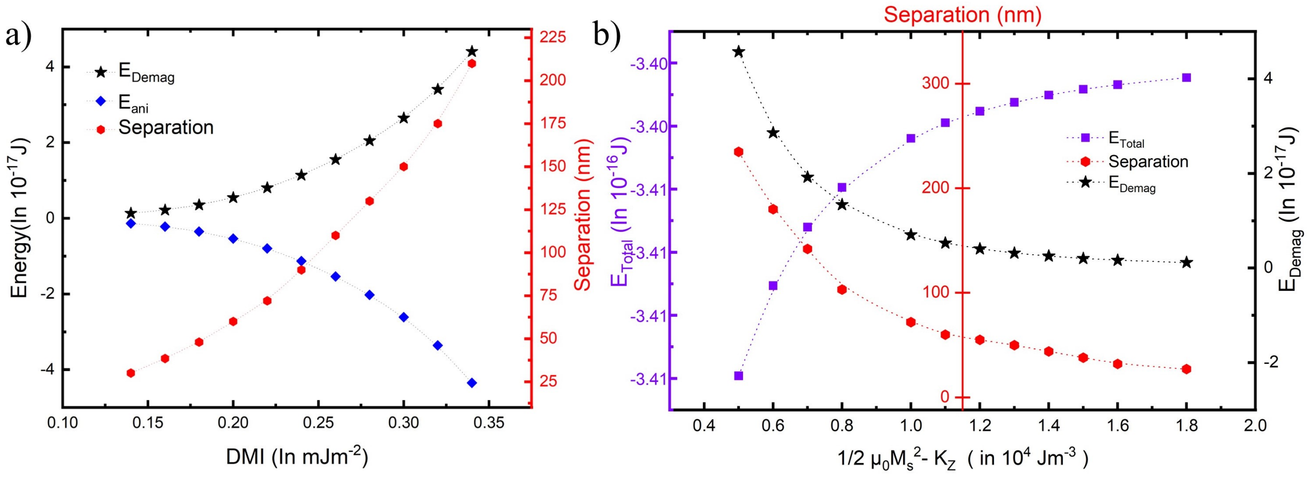

Figure S8. Evolution of the separation between meron-antimeron and the energy terms, including total energy (E_{total}), and demag energy (E_{demag}), and aniostropy (E_{ani}) energy as a function of (a) DMI, and (b) effective anisotropy ($|K_{\text{eff}}|$). The results highlight the critical role of DMI and perpendicular magnetic anisotropy in stabilizing the bimeron near the spin reorientation transition (SRT) region.
